# Magnetic field-dependent spin structures of nanocrystalline holmium

**DOI:** 10.1107/S1600576716001898

**Published:** 2016-03-08

**Authors:** Philipp Szary, Daniel Kaiser, Jens-Peter Bick, Dieter Lott, André Heinemann, Charles Dewhurst, Rainer Birringer, Andreas Michels

**Affiliations:** a Physics and Materials Science Research Unit, University of Luxembourg, 162A Avenue de la Faïencerie, L-1511 Luxembourg, Luxembourg; b Zentrum für Material- und Küstenforschung GmbH, Helmholtz-Zentrum Geesthacht, Max-Planck-Straße 1, D-21502 Geesthacht, Germany; c Large Scale Structures Group, Institut Laue–Langevin, 71 Avenue des Martyrs, SB 335, F-38042 Grenoble, France; d Experimentalphysik, Universität des Saarlandes, D-66041 Saarbrücken, Germany

**Keywords:** neutron diffraction, spin structures, rare earth metals, magnetic materials, nanocrystalline holmium

## Abstract

The results are reported of magnetic field-dependent neutron diffraction experiments on polycrystalline inert-gas condensed holmium with a nanometre crystallite size (*D* = 33 nm). At *T* = 50 K, no evidence is found for the existence of helifan(3/2) or helifan(2) structures for the nanocrystalline sample, in contrast with results reported in the literature for the single crystal. Instead, when the applied field *H* is increased, the helix pattern transforms progressively, most likely into a fan structure. It is the component of *H* which acts on the basal-plane spins of a given nanocrystallite that drives the disappearance of the helix; for nanocrystalline Ho, this field is about 1.3 T, and it is related to a characteristic kink in the virgin magnetization curve. For a coarse-grained Ho sample, concomitant with the destruction of the helix phase, the emergence of an unusual angular anisotropy (streak pattern) and the appearance of novel spin structures are observed.

## Abstract 2

Neutron diffraction experiments on nanocrystalline holmium suggest the absence of helifan(3/2) and helifan(2) structures at 50 K.

## 1. Introduction   

While the spin structures of most of the heavy rare earth metals are well understood in zero magnetic field (Koehler, 1965 ▸), the situation becomes more complicated once an external magnetic field is applied (Jensen & Mackintosh, 1991 ▸). This is particularly so for the case of holmium (Koehler *et al.*, 1966 ▸), where the application of a field (Koehler *et al.*, 1967 ▸) results in the emergence of a number of complicated magnetic structures, *e.g.* the so-called helifan phases (see below) (Jensen, 1996 ▸). In general, the respective ground-state spin configuration at a certain temperature reflects the delicate interplay between various magnetic energy terms, such as the Zeeman energy, the magnetodipolar interaction, the exchange energy, and the magnetocrystalline and magneto­elastic anisotropy.

Most of the experimental and theoretical studies that have so far been carried out on Ho [which crystallizes in the hexagonal close-packed (h.c.p.) structure] refer to the ideal­ized imperfection-free single-crystalline reference state. In zero field, Ho exhibits an antiferromagnetic helical phase between the Néel temperature *T*_N_ = 132 K and the Curie point *T*_C_ = 20 K (Legvold, 1980 ▸); the spins are then ferromagnetically aligned within the basal planes of the h.c.p. lattice (*b* axis = easy axis) and turn by a certain angle from layer to layer. For single-crystal Ho, *a* = 3.5778 Å and *c* = 5.6178 Å at room temperature (Legvold, 1980 ▸). The resulting helix is described by an ordering wavevector **q**, which decreases monotonically in magnitude between ∼0.28 (in units of 2π/*c*) at *T*_N_ to 0.167 ≃ 1/6 at *T*_C_ (Jensen, 1996 ▸). Below about 20 K, a ferromagnetic component develops along the *c* axis, giving rise to a conical phase.

The effect of a magnetic field applied in the basal plane of Ho has been studied extensively by Jensen & Mackintosh (1990 ▸). Above about 40 K, where the crystal-field anisotropy is small compared with the exchange energy, those authors predicted the appearance of various helifan phases, intermediate structures between a helix and a fan, which result from the competition between the applied field (favouring complete alignment of magnetic moments) and the exchange interaction (aiming to establish periodic structures). The calculations of Jensen and Mackintosh were then able to explain the early work of Koehler *et al.* (1967 ▸), and single-crystal neutron diffraction data (Jehan *et al.*, 1992 ▸; Kosugi *et al.*, 2003 ▸) proved the existence of the helifan(3/2) and helifan(2) phases. Specifically, at 50 K, Kosugi *et al.* (2003 ▸) found the sequence of structures helix 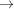 helifan(3/2) 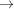 helifan(2) 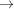 fan when the field (applied along the *b* axis) was increased from zero to 2.2 T.

Our aim is to investigate the impact of poly/nano­crystallinity on the field-dependent spin microstructure of Ho metal at a temperature of 50 K, where a large body of both experimental and theoretical data exists (in particular regarding helifan structures). As is well known, the structural atomic site disorder is related to the large interface-to-volume ratio in polycrystalline nanomagnets. With a typical average crystallite size of *D* ≃ 10 nm, it couples to the spin distribution by virtue of the magnetoelastic energy and results in an associated spin disorder at or across internal interfaces (grain boundaries). Note that the volume fraction of internal interfaces scales as *D* ^−1^ [assuming a grain-boundary thickness of δ = 1 nm and a lognormal grain-size distribution of typical width σ = 1.7, the volume fraction of grain boundaries in a nanocrystalline inert-gas condensed material with a volume-weighted average grain size *D* (measured by X-ray diffraction) can be roughly estimated as 4δ/*D* (Krill & Birringer, 1998 ▸; Döbrich *et al.*, 2012 ▸)]. Likewise, disordered grain-boundary regions may also alter the highly position-sensitive Rudermann–Kittel–Kasuya–Yosida exchange interaction, which couples the strongly localized 4*f* moments in the rare earth metals involving conduction electrons. Indeed, several investigations of nanocrystalline and nanoscaled rare earth metals (O’Shea & Perera, 1999 ▸; Michels *et al.*, 2002 ▸, 2008 ▸, 2011 ▸; Yan *et al.*, 2003 ▸; Weissmüller *et al.*, 2004 ▸; Kruk *et al.*, 2006 ▸; Yue *et al.*, 2006 ▸, 2008 ▸; Philippi *et al.*, 2009 ▸; Döbrich *et al.*, 2012 ▸; Ryan *et al.*, 2013 ▸; Ferdinand *et al.*, 2014 ▸) have provided compelling evidence that a reduced particle size has a strong impact on magnetic properties such as coercivity, approach-to-saturation behaviour, Curie transition temperature and easy-axis canting angle.

Since the mean-field calculations of Jensen & Mackintosh (1991 ▸) reveal – for a defect-free single crystal – that at 50 K the different magnetic phases of Ho lie energetically close together when the applied field is varied, one may expect that polycrystallinity alters the energy scale in the material, resulting in different magnetic ground states. In this paper, we report the results of magnetic field-dependent neutron diffraction experiments along the (000) forward direction on a nanocrystalline and a coarse-grained Ho sample at 50 K; for a recent magnetization and specific heat study of single-crystalline Ho, see Zverev *et al.* (2015 ▸).

## 2. Experimental   

**Figure 1:**
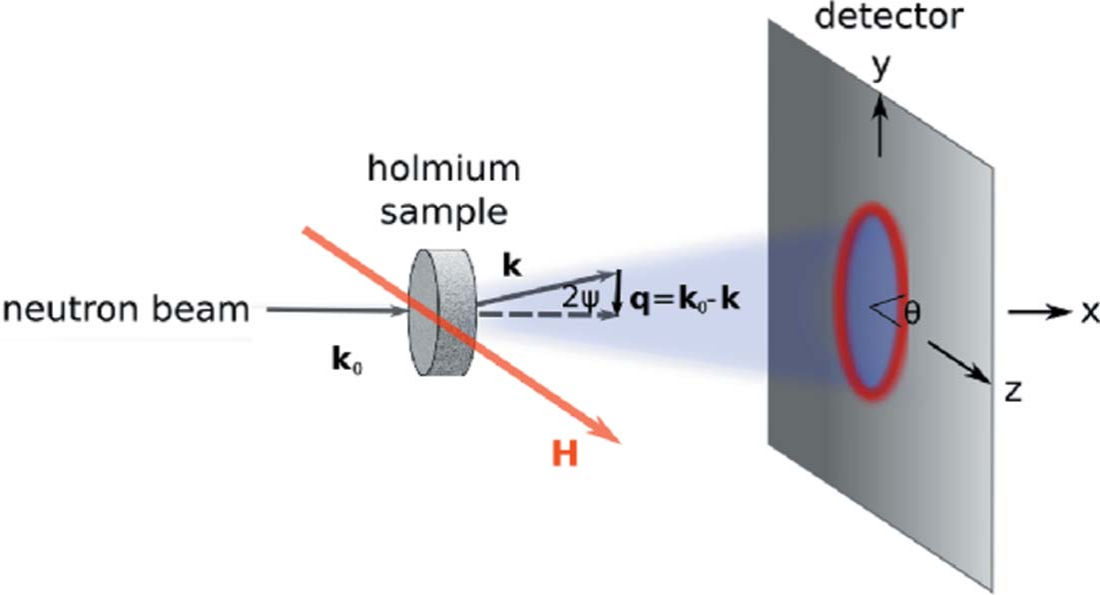
A sketch of the neutron scattering setup. The wavevectors of the incident and scattered neutrons are, respectively, denoted **k**_0_ and **k**. The angle θ is measured between the applied-field direction **H** || **e**_*z*_ and the scattering vector **q** = **k**_0_ − **k** ≃ (0, *q_y_*, *q_z_*); |**q**| = *q* = 4πλ^−1^sinψ, where λ is the average neutron wavelength and 2ψ is the scattering angle.

Nanocrystalline bulk Ho samples were prepared by inert-gas condensation and subsequent compaction (Birringer, 1989 ▸; Michels *et al.*, 2002 ▸; Weissmüller *et al.*, 2004 ▸; Döbrich *et al.*, 2012 ▸). The as-prepared specimens had the shape of a circular disc (pellet) with a diameter of 8 mm and a thickness of typically 0.5 mm. We note that the crystallites were not able to rotate during the neutron experiment, *e.g.* when applying the magnetic field. One as-prepared sample was annealed under He atmosphere at 973 K for 4 h in order to induce grain growth and obtain a micrometre-sized coarse-grained microstructure. Unpolarized neutron scattering experiments were carried out on the instrument SANS 1 at the Forschungs-Neutronenquelle Heinz Maier-Leibnitz (FRM II), Garching, Germany, and on beamline D33 at the Institut Laue–Langevin, Grenoble, France. A sketch of the measurement setup is shown in Fig. 1 ▸.

All neutron data were collected in transmission geometry with the applied magnetic field **H** perpendicular to the wavevector **k**_0_ of the incoming neutron beam. For the neutron measurements at 50 K, the sample was cooled in zero field from 300 K through *T*_N_ ≃ 132 K to 50 K, where Ho is in the antiferromagnetic helical state. Two-dimensional detector images were taken at different field values, following the course of the virgin magnetization curve. Neutron raw data were corrected for background scattering (empty sample holder), transmission and detector efficiency. The mean wavelength of the neutrons was set to λ = 4.67 Å with Δλ/λ ≃ 10% (full width at half-maximum, FWHM). Further sample characterization was carried out by means of wide-angle X-ray diffraction (grain-size and microstrain determination) and AC/DC magnetization measurements.

## 3. Results and discussion   

### 3.1. X-ray diffraction and magnetometry   

**Figure 2:**
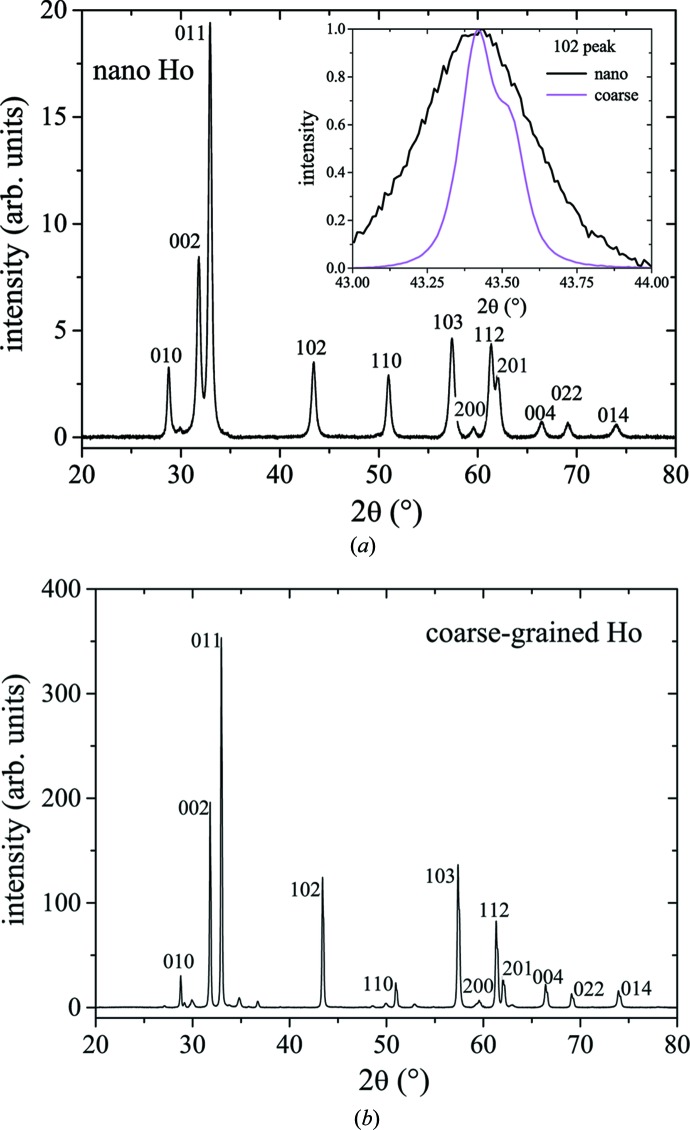
X-ray diffraction scans (using Cu *K*α_1,2_ radiation) of (*a*) as-prepared inert-gas condensed nanocrystalline Ho and (*b*) coarse-grained Ho. The inset in (*a*) compares the normalized 102 peak of nanocrystalline Ho with that of the coarse-grained Ho sample; the shoulder in the data of the coarse-grained sample is due to the *K*α_2_ peak.

**Figure 3:**
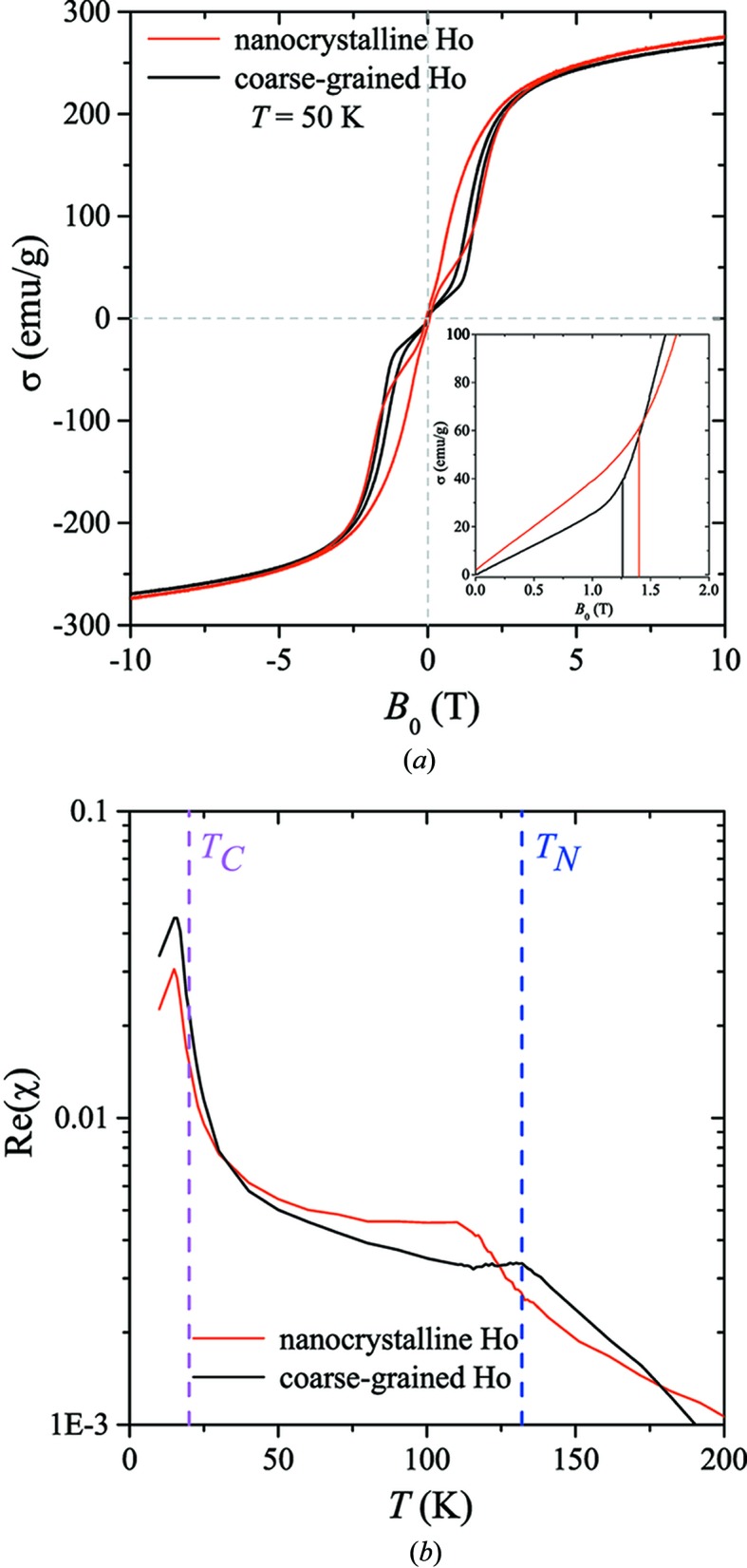
(*a*) Magnetic hysteresis loops for nanocrystalline and coarse-grained Ho measured at 50 K after zero-field cooling. The inset displays the recorded virgin curves; note the kinks at ∼1.40 T (nanocrystalline Ho) and ∼1.26 T (coarse-grained Ho). (*b*) The temperature dependence of the real part of the AC susceptibility of nanocrystalline and coarse-grained Ho (log-linear scale). The vertical magenta and blue dashed lines indicate, respectively, the Curie (*T*_C_) and Néel (*T*_N_) temperature of bulk Ho.

Fig. 2 ▸ displays X-ray diffractograms of nanocrystalline and coarse-grained Ho. The observed narrowing of the Bragg peaks (see inset in Fig. 2 ▸ *a*) is related to the annealing-induced increase of the average crystallite size *D* and to the reduction of the microstrain level *e*. Analysis of the X-ray data according to the method of Klug & Alexander (1974 ▸) yields *D* = 33 ± 3 nm and *e* ≃ 0.23% for the nanocrystalline sample. The coarse-grained Ho sample (Fig. 2 ▸ *b*) could not be analysed, since the measured Bragg-peak widths are comparable to the instrumental resolution of the X-ray diffractometer, which indicates a coarse-grained microstructure (*D* ≳ 100 nm). The diffractogram of the coarse-grained sample additionally contains small impurity peaks, presumably due to oxide and nitride compounds at the sample surface. Measurements of the *M*(*H*) magnetic hysteresis and of the temperature dependence of the AC susceptibility of nanocrystalline and coarse-grained Ho are shown in Fig. 3 ▸.

Both Ho samples were cooled in zero magnetic field from the paramagnetic state at room temperature through *T*_N_ to 50 K, in agreement with the protocol in the neutron experiment. As expected, because of the extremely large magnetic anisotropy of Ho, neither sample can be saturated by the available maximum applied field of 10 T (Fig. 3 ▸ *a*). However, the reversible regime of the hysteresis loop can be reached already at field values of μ_0_ *H* ≳ 4 T. This observation shows that the maximum field available in the neutron experiment (μ_0_ *H*_max_ = 5 T) is sufficient to bring the samples into the reversible regime (major loop). The virgin curves of both samples (see inset in Fig. 3 ▸ *a*) reveal characteristic kinks, *i.e.* deviations from quasi-linearity at applied fields of ∼1.40 T for nanocrystalline Ho and ∼1.26 T for coarse-grained Ho. As we will see below, these features can be related to the destruction of the antiferromagnetic helix with increasing field.

The AC-susceptibility data (Fig. 3 ▸ *b*) reveal a significantly lower Néel point of ∼113 K for nanocrystalline Ho, compared with the coarse-grained sample which peaks at the literature value of *T*_N_ = 132 K (Legvold, 1980 ▸). A grain-size dependence of a critical point has previously been observed in Gd, Tb and Dy (O’Shea & Perera, 1999 ▸; Michels *et al.*, 2002 ▸, 2011 ▸; Yue *et al.*, 2006 ▸, 2008 ▸; Philippi *et al.*, 2009 ▸; Ferdinand *et al.*, 2014 ▸). Also in agreement with previous results (Michels *et al.*, 2011 ▸), the Curie temperatures of the two samples are very close to each other and close to the literature value.

### 3.2. Neutron diffraction   

**Figure 4:**
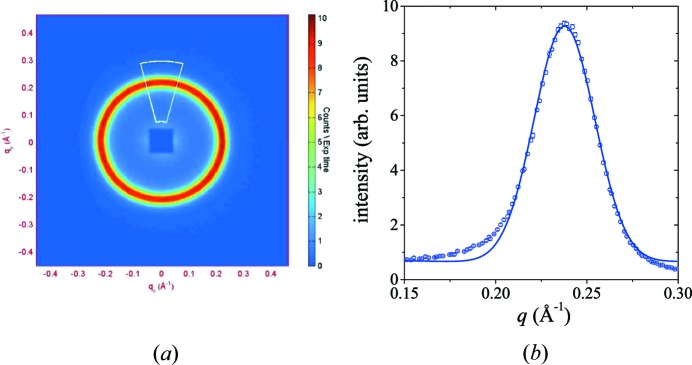
(*a*) Neutron diffraction pattern of nanocrystalline (*D* = 33 nm) Ho at 50 K after ZFC. The white line marks the direction along which the data have been azimuthally averaged (± 12.5° sector average). (*b*) Azimuthally averaged neutron intensity as a function of momentum transfer *q*. Owing to the wavelength spread of the incident neutrons, Δλ/λ ≃ 10%, the *q* resolution at the peak position (q* ≃ 0.24 Å^−1^) is about Δ*q* ≃ 0.01 Å^−1^ (Pedersen *et al.*, 1990 ▸).

Fig. 4 ▸(*a*) displays for nanocrystalline Ho the distribution of scattered neutrons on the two-dimensional position-sensitive detector, measured at 50 K after zero-field cooling (ZFC) through *T*_N_. The observed Bragg peak (Debye–Scherrer ring) can be identified by magnetic Bragg diffraction on the antiferromagnetic helix with a modulation vector magnitude of *q* ≃ 0.213 (in units of 2π/*c*). The azimuthally averaged (over 2π) data (Fig. 4 ▸ *b*) allow us to determine the wavevector magnitude of the helix by fitting a combination of Lorentzians and Gaussians to the measured data (solid line in Fig. 4 ▸ *b*); ± 12.5° sector averages along the vertical and horizontal direction have also been computed (Fig. 4 ▸ *a*). For the sake of clarity, we will in the following neglect in all two-dimensional neutron intensity graphs the colour scale for the intensity and the scales for the *q* axes. Likewise, we denote with **q** interchangeably the scattering vector and the modulation vector.

**Figure 5:**
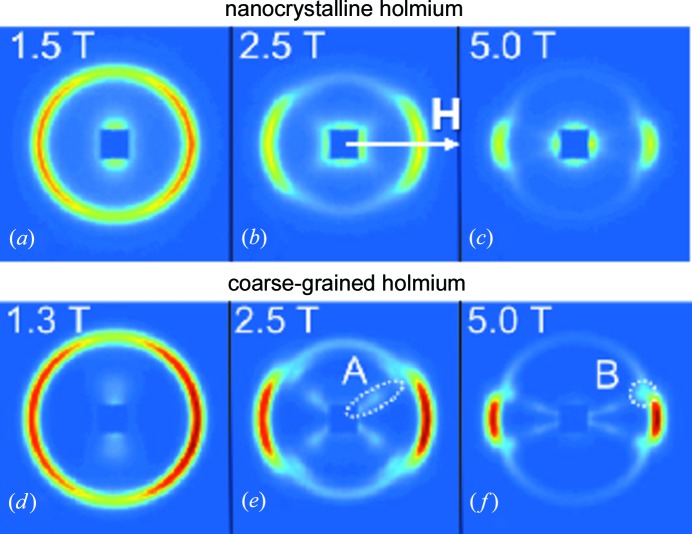
Magnetic field-dependent two-dimensional diffraction patterns of (*a*)–(*c*) nanocrystalline (*D* = 33 nm) and (*d*)–(*f*) coarse-grained (*D* ≳ 100 nm) Ho at 50 K after ZFC. **H** is horizontal in the plane of the detector and increases up to 5 T (virgin-curve data). The central square in each figure masks the region of the beam stop. The white dashed circles labelled ‘A’ and ‘B’ refer to features discussed in the main text.

**Figure 6:**
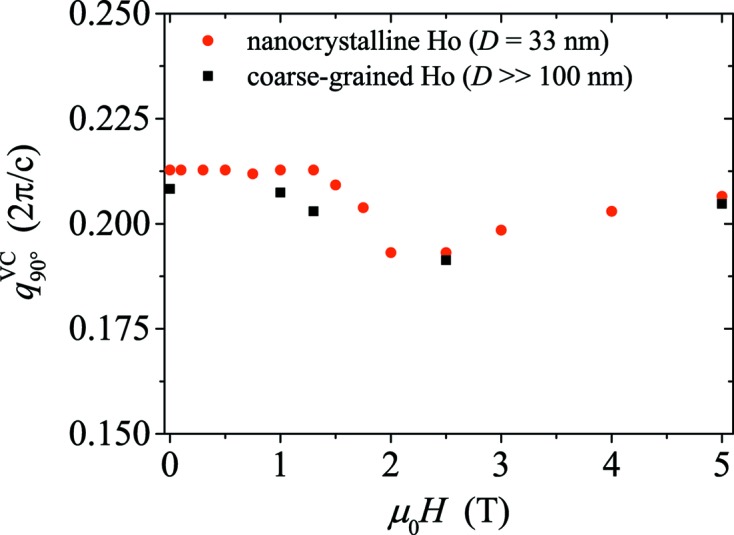
The field dependence of the wavevector magnitude (in units of 2π/*c*) in the direction normal to **H** for nanocrystalline and coarse-grained Ho.

The evolution of the helix pattern with increasing field (up to 5 T) is depicted in Fig. 5 ▸ for nanocrystalline and coarse-grained Ho. Qualitatively, both samples exhibit the same behaviour: the helix is stable up to a field of at least 1.0 T, as shown by homogeneous diffraction rings (data not shown) similar to the one shown in Fig. 4 ▸(*a*). For larger fields, the helix-peak intensity decreases in the direction perpendicular to the field and at 5 T is concentrated in the directions parallel and antiparallel to **H**. Fig. 6 ▸ displays the field dependence of the wavevector magnitude in the direction normal to the field, 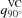 (VC = virgin curve); 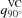 is constant up to ∼1.3 T for nanocrystalline Ho and up to ∼1.0 T for coarse-grained Ho, then drops slightly, and above 2 T exhibits a tendency to increase again. The modulation vector along the horizontal direction (θ = 0°) is field independent for both samples with 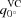 ≃ 0.212 for nanocrystalline Ho and 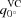 ≃ 0.208 for coarse-grained Ho (data not shown).

In the inert-gas condensation process, nanometre-sized Ho clusters (which condense from the metal vapour) are collected on a liquid-nitrogen-cooled cold finger and compacted under high vacuum at GPa pressures (Birringer, 1989 ▸; Michels *et al.*, 2002 ▸; Weissmüller *et al.*, 2004 ▸; Döbrich *et al.*, 2012 ▸). As a consequence, in inert-gas condensed (powder) samples, the set of crystallographic easy axes for the magnetization changes randomly at each grain boundary. The random orientation of the crystallites and the associated random variation of the modulation vectors **q** is shown by the observation of a homogeneous Debye–Scherrer ring (compare *e.g.* Fig. 4 ▸ *a*). Regarding the field variation of the diffraction pattern, this implies the following: those nanocrystals with their *c* axis (= propagation direction of **q**) aligned perpendicularly to **H** experience the full magnitude of the external field within the basal planes. Since the in-plane magnetic anisotropy of Ho is much smaller than the out-of-plane magnetic anisotropy (Jensen & Mackintosh, 1991 ▸; Welsch *et al.*, 2005 ▸), an increasing horizontal field rotates the magnetic moments of successive lattice planes along its direction, in this way progressively ‘destroying’ the helical structure. This effect is strongest for ‘θ = 90° oriented’ and weakest for ‘θ = 0° oriented’ grains (for the definition of the angle θ see Fig. 1 ▸), provided that *H* remains much smaller than the out-of-plane anisotropy field. Hence, one observes a successive decrease of the magnetic Bragg intensity from 90° towards the horizontal direction (compare *e.g.* Figs. 5 ▸ *a*–5 ▸ *c*).

*Ab initio* density functional theory calculations (Welsch *et al.*, 2005 ▸) indicate that, for bulk Ho, the *c* axis crystal-field parameter is an order of magnitude larger than the in-plane parameter. This result supports our assumption made above that an applied field of the order of 5 T is able to rotate the magnetic moments inside the basal plane but is insufficient to produce a significant out-of-plane tilting. Moreover, the computations of Welsch *et al.* (2005 ▸) also predict nonzero crystal-field parameters *A*_43_ and *A*_63_, which originate from symmetry reduction at surfaces. Such terms may play a particularly important role in nanocrystalline Ho, where a significant number of atoms are located in the near vicinity of grain boundaries. As a consequence, novel magnetic ground states may appear or well known spin configurations may not be visible. In fact, in the neutron data for the nanocrystalline Ho sample we find no evidence for the existence of the helifan(3/2) or helifan(2) structures, which were previously observed in Ho single crystals for fields below ∼2.0 T (Jehan *et al.*, 1992 ▸; Kosugi *et al.*, 2003 ▸); at 50 K and for fields between 1.2 and 2.0 T, these structures would, respectively, give rise to modulation vector magnitudes of *q* ≃ 0.135 and *q* ≃ 0.095 (Kosugi *et al.*, 2003 ▸). This observation may appear surprising, since a nanocrystal with a size of 33 nm contains about 120 hexagonal layers; taking into account that at 50 K the helifan(3/2) structure has a period of about 15 layers, a coherence length of eight times one period may be considered sufficient for establishing a well defined structure.

In addition to magnetic Bragg diffraction, we observe in Fig. 5 ▸ that, with increasing field, a diffuse ferromagnetic scattering contribution builds up at small *q* around the beamstop. The diffuse magnetic small-angle scattering is larger in the nanocrystalline sample owing to nanometre-scale magnetization fluctuations. Obviously, nuclear small-angle scattering, which is field independent and isotropic for this material, is weak for both samples. In particular, for the coarse-grained sample we also observe (with increasing *H*) an interesting angular anisotropy with four streaks (labelled ‘A’ in Fig. 5 ▸ *e*). Above about 2.0 T, this feature is also present (although less pronounced) in the data from the nano­crystalline sample. The angle θ_crit_ that these streaks form with the horizontal direction is field dependent and decreases with increasing field; the streak pattern seems to be correlated with the helix transition. From the observation that the intensity of such a streak appears to extend over a rather broad range of momentum transfers, one may conclude that the origin of the streaks is possibly related to a diffuse magnetization distribution, rather than to long-range periodic structures. However, a closer inspection of the coarse-grained Ho data at 2.5 and 5 T (Figs. 5 ▸ *e* and 5 ▸ *f*, respectively) might indicate the existence of a slight modulation in the streak pattern; such a modulation is absent in the nanocrystalline sample (data not shown). Even more puzzling is the appearance of four additional Bragg peaks (labelled ‘B’ in Fig. 5 ▸ *f*) in the same direction as the streak pattern. The origin of the streaks and their modulation may indeed be related to a periodic structure; however, the potential modulation seems to be significantly different, as would be expected for a helifan(3/2) or helifan(2) structure. The clarification of this question will be the subject of further investigations.

**Figure 7:**
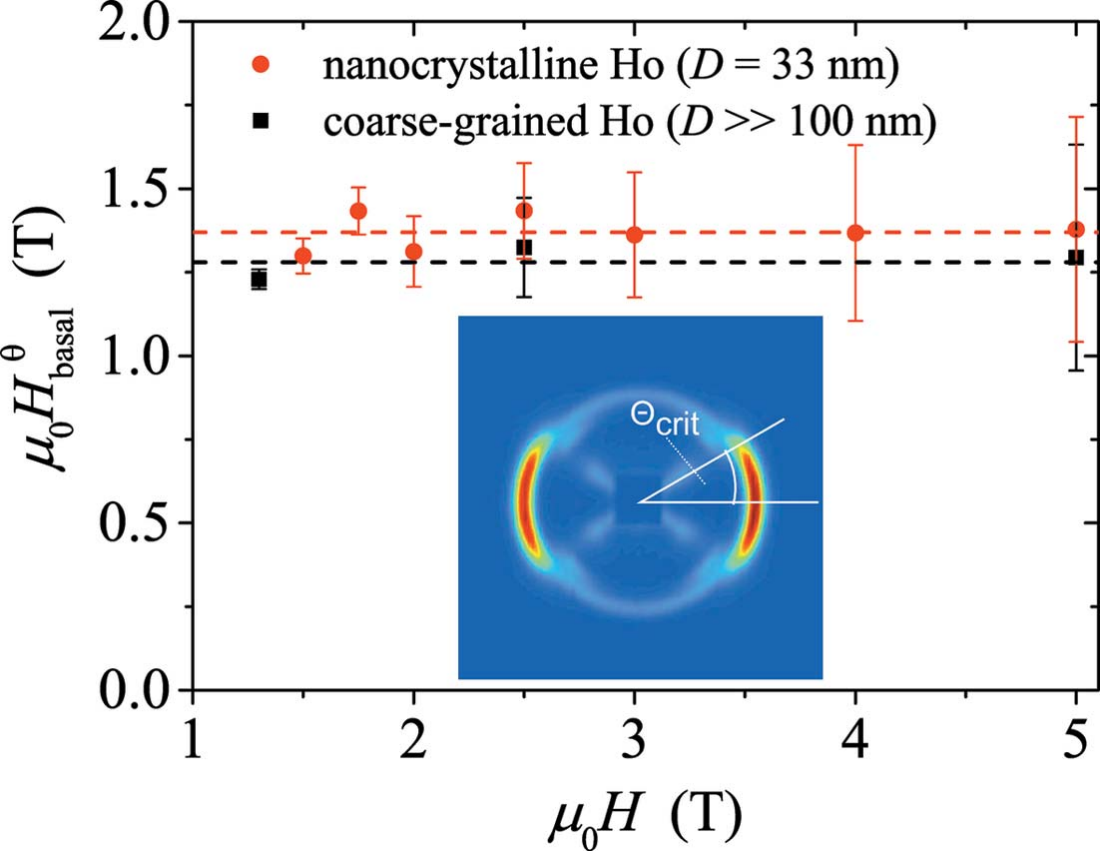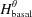 = *H*sinθ_crit_ for nanocrystalline and coarse-grained Ho. 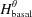 corresponds to the projection of the applied field into the basal plane of a crystallite which has its *c* axis oriented in the direction θ_crit_ (see inset).

The disappearance of the helical pattern can be evaluated more quantitatively (and related to features of the DC magnetization curve) by considering the projections of the applied field **H** along the *c* axis and within the basal plane of a given crystallite (domain) which has its *c* axis oriented along the direction **q** = (*q*, θ). In our scattering geometry, only those components of the scattering vector **q** that are normal to the incident neutron beam (**k**_0_) are probed effectively. For **k**_0_ || **e**_*x*_ and **H** || **e**_*z*_ (compare Fig. 1 ▸), we have **q**/*q* = (0, sinθ, cosθ), and the projections of **H** along the *c* axis and the basal plane of a crystal are, respectively, given by 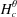 ≃ *H*cosθ and 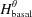 ≃ *H*sinθ. In Fig. 7 ▸, we plot 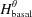 = *H*sinθ_crit_, where θ_crit_ denotes the angle between **H** and the streak pattern (see Fig. 7 ▸). It is seen that (within experimental uncertainty) the basal-plane-projected fields take on constant values of 1.37 ± 0.20 T for nanocrystalline Ho and 1.27 ± 0.20 T for coarse-grained Ho. Around these field values, kinks in the DC magnetization curves occur, as shown in the inset of Fig. 3 ▸(*a*). These values also lie within the range of the reported transition fields for the phase transition from helix to helifan(3/2) in the single crystal (Jehan *et al.*, 1992 ▸; Kosugi *et al.*, 2003 ▸). However, since we do not observe the helifan(3/2) [or helifan(2)] structure in our neutron data from the nanocrystalline Ho sample, one may ask to which spin configuration the helix evolves. By taking a look at the single-crystal phase diagram (Jensen & Mackintosh, 1990 ▸), it is evident that the fan structure would be a suitable candidate, since it is characterized by a smaller intensity and by a wavevector that is only slightly smaller than the modulation vector of the helix, in agreement with the observations in Figs. 5 ▸ and 6 ▸. For the coarse-grained Ho sample, the occurrence of the streak pattern with its slight modulation indicates that, around the critical field, a periodic structure may form before the transition into a fan structure at higher magnetic fields.

## 4. Summary and conclusions   

At *T* = 50 K, we have studied by means of neutron diffraction along the (000) forward direction the magnetic field-dependent spin structure of polycrystalline inert-gas condensed Ho with an average crystallite size of *D* = 33 nm. As the applied magnetic field **H** is increased from zero to 5 T, we observe that the Debye–Scherrer ring, which is characteristic for magnetic neutron diffraction on the antiferromagnetic helix, ‘breaks up’ in the direction perpendicular to **H** and that the scattering intensity becomes progressively concentrated along the direction parallel to **H**. It is the projected field component into the basal plane of a given domain (crystallite) which drives the transition from the helical state to a periodic ordering (*e.g.* fan structure) with a large ferromagnetic component; the critical field value of ∼1.3–1.4 T where the helix is destroyed appears to be unique and is related to a characteristic kink in the virgin magnetization curve. In contrast with results obtained on the Ho single crystal where helifans were observed below about 2.0 T, we find no evidence for the existence of helifan(3/2) or helifan(2) structures in nanocrystalline Ho; instead, our data are indicative of a direct helix 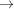 fan transition. In a coarse-grained Ho sample, we observe with increasing field the build-up of a slightly modulated magnetic scattering intensity (streak pattern), which is correlated with the vanishing of the helix diffraction pattern and may be an indication of the occurrence of a long-period magnetic structure. Our results demonstrate that polycrystallinity has a strong impact – presumably due to associated changes in the energy scale of the material – on the ground-state spin configuration of Ho at 50 K. Therefore, it would be of interest to include the presumed effects of polycrystallinity (*e.g.* a distribution of exchange-interaction strengths, crystal-field parameters related to the symmetry reduction at interfaces, crystallite-size distribution) into a theoretical description of the problem. From an experimental point of view, the use of spin-polarized neutrons (Honecker *et al.*, 2010 ▸; Michels, 2014 ▸) might provide a means of obtaining further insights into the field-dependent spin structures of Ho.
